# Assessing temporal differences in the predictive power of baseline TyG-related parameters for future diabetes: an analysis using time-dependent receiver operating characteristics

**DOI:** 10.1186/s12967-023-04159-7

**Published:** 2023-05-04

**Authors:** Maobin Kuang, Ruijuan Yang, Xin Huang, Chao Wang, Guotai Sheng, Guobo Xie, Yang Zou

**Affiliations:** 1 Medical College of Nanchang University, Nanchang, 330006 Jiangxi China; 2 Department of Cardiology, Jiangxi Provincial People’s Hospital, The First Affiliated Hospital of Nanchang Medical College, Nanchang, 330006 Jiangxi China; 3 Jiangxi Cardiovascular Research Institute, Jiangxi Provincial People’s Hospital, The First Affiliated Hospital of Nanchang Medical College, Nanchang, 330006 Jiangxi China; 4 Department of Endocrinology, Jiangxi Provincial People’s Hospital, The First Affiliated Hospital of Nanchang Medical College, Nanchang, 330006 Jiangxi China

**Keywords:** TyG index, TyG-related parameters, Diabetes, Time-dependent ROC analysis, Prediction

## Abstract

**Background:**

It is known that measuring the triglyceride glucose (TyG) index and TyG-related parameters [triglyceride glucose-body mass index (TyG-BMI), triglyceride glucose-waist circumference (TyG-WC), and triglyceride glucose-waist to height ratio (TyG-WHtR)] can predict diabetes; this study aimed to compare the predictive value of the baseline TyG index and TyG-related parameters for the onset of diabetes at different future periods.

**Methods:**

We conducted a longitudinal cohort study involving 15,464 Japanese people who had undergone health physical examinations. The subject’s TyG index and TyG-related parameters were measured at the first physical examination, and diabetes was defined according to the American Diabetes Association criteria. Multivariate Cox regression models and time-dependent receiver operating characteristic (ROC) curves were constructed to examine and compare the risk assessment/predictive value of the TyG index and TyG-related parameters for the onset of diabetes in different future periods.

**Results:**

The mean follow-up period of the current study cohort was 6.13 years, with a maximum of 13 years, and the incidence density of diabetes was 39.88/10,000 person-years. In multivariate Cox regression models with standardized hazard ratios (HRs), we found that both the TyG index and TyG-related parameters were significantly and positively associated with diabetes risk and that the TyG-related parameters were stronger in assessing diabetes risk than the TyG index, with TyG-WC being the best parameter (HR per SD increase: 1.70, 95% CI 1.46, 1.97). In addition, TyG-WC also showed the highest predictive accuracy in time-dependent ROC analysis for diabetes occurring in the short-term (2–6 years), while TyG-WHtR had the highest predictive accuracy and the most stable predictive threshold for predicting the onset of diabetes in the medium- to long-term (6–12 years).

**Conclusions:**

These results suggest that the TyG index combined with BMI, WC, and WHtR can further improve its ability to assess/predict the risk of diabetes in different future periods, where TyG-WC was not only the best parameter for assessing diabetes risk but also the best risk marker for predicting future diabetes in the short-term, while TyG-WHtR may be more suitable for predicting future diabetes in the medium- to long-term.

**Supplementary Information:**

The online version contains supplementary material available at 10.1186/s12967-023-04159-7.

## Background

Diabetes is a metabolic disease caused by insulin insensitivity, insulin deficiency, and impaired biological function due to genetic and environmental factors [1]. In the past few decades, the prevalence of diabetes worldwide has risen sharply with the aging of the population, great changes in dietary patterns and lifestyles, and the prevalence of obesity, and it has become a serious global health problem, causing a huge economic burden (diabetes-related economic expenditures account for approximately 10 percent of global health expenditures) [2, 3]. Therefore, the prevention of diabetes has become a global public health priority with significant practical implications [4, 5].

Insulin resistance (IR) is a state of reduced responsiveness of target cells or the whole organism to the insulin concentrations to which they are exposed, usually preceding the onset of diabetes, and has been identified as a key mediator of diabetes as well as cardiovascular disease [6–8]. The hyperinsulinemic-euglycemic clamp is the gold standard for measuring IR, but it is not suitable for clinical practice due to its invasive and complicated examination process [9]. Homeostatic model assessment of insulin resistance (HOMA-IR) is currently the most widely used non-invasive measurement approach in clinical practice, but the application of this approach in patients with impaired β-cell function and insulin therapy is not ideal [10]. To address these limitations, the TyG index was developed, which consists only of triglycerides (TG) and fasting plasma glucose (FPG) and does not require quantitative insulin measurements nor is it affected by insulin therapy; in addition, the TyG index is easily accessible in epidemiological studies and clinical practice and is superior to HOMA-IR in identifying IR in the general population [11–13]. In this context, a large number of clinical studies have explored the TyG index in-depth, and a growing body of evidence suggested that the TyG index has great application value in assessing/predicting the risk of diabetes, cardiovascular disease, and other metabolic diseases [14–17]. Furthermore, in recent years, many researchers have found that combining TyG index and obesity parameters (BMI, WC, and WHtR) into TyG-related parameters can further improve the ability to identify IR [18, 19]; and the latest research also found that TyG-related parameters were generally better than the TyG index in identifying fatty liver and predicting the progression of coronary artery calcification [20, 21]. However, there is no conclusive evidence as to the superiority of the TyG index and TyG-related parameters in assessing the risk of diabetes and in predicting the onset of diabetes in different future periods. To clarify the answer to these questions, this study compared the risk assessment/predictive value of the TyG index and TyG-related parameters for diabetes by standardizing the HRs of the TyG index and TyG-related parameters associated with diabetes risk and constructing time-dependent ROC curves at different time points to calculate the corresponding area under the curves (AUCs) and thresholds.

## Methods

### Study data and population

**Fig. 1:**
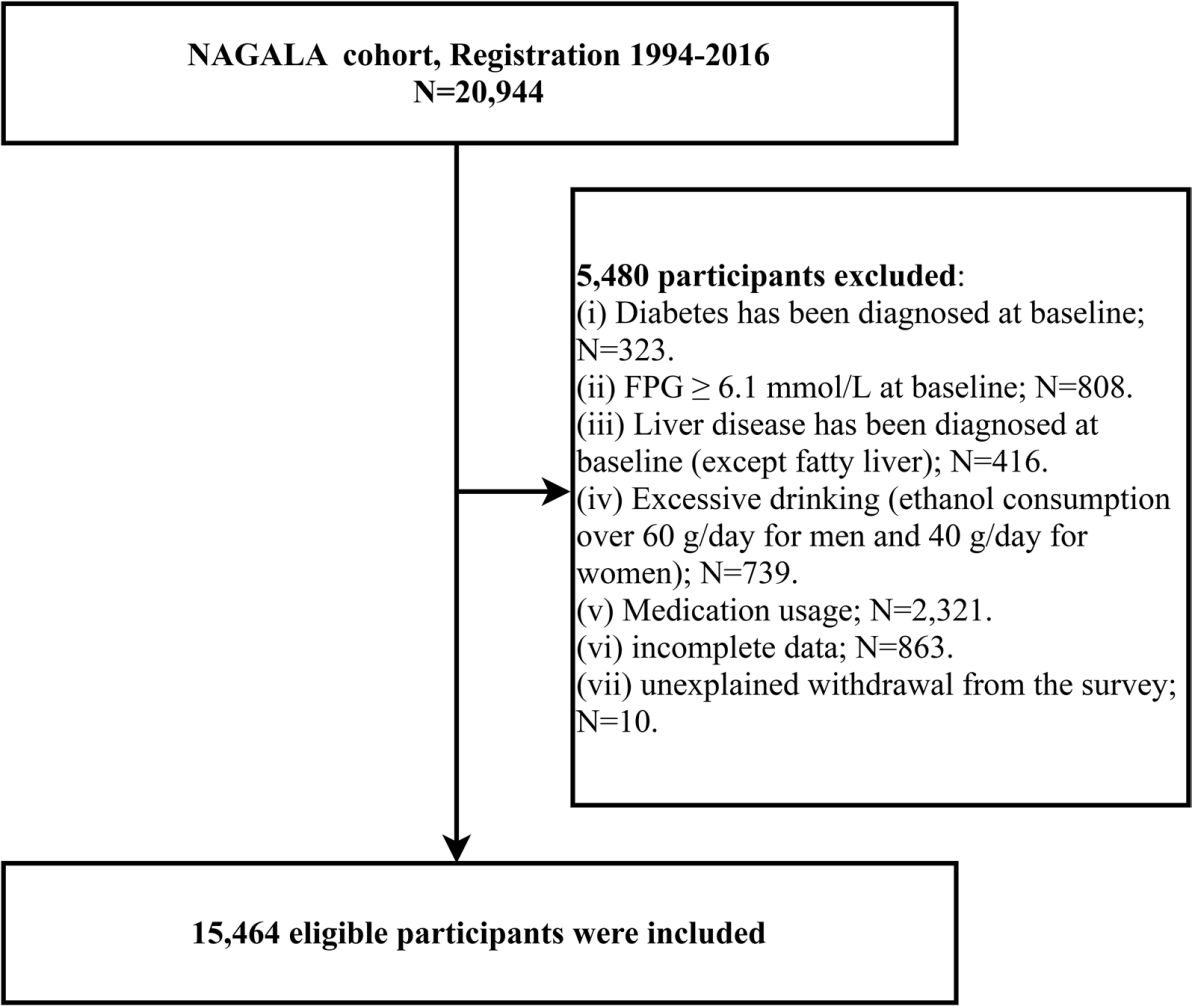
Flowchart of the selection process of study subjects

We used data from the NAGALA (NAfld in the Gifu Area, Longitudinal Analysis) cohort study. This cohort has been continuously recruiting people who have attended health checkups at the Murakami Memorial Hospital since 1994, aiming to prospectively investigate the incidence and related factors of chronic diseases such as diabetes and nonalcoholic fatty liver disease. The cohort has been described in detail in a previous study by Okamura et al. [22], and the available dataset of the study cohort has been uploaded to the Dryad public database for sharing [23]. In the current study, we included the data of 20,944 subjects who were registered in the NAGALA cohort from 1994 to 2016. According to the purpose of this study, we excluded subjects with diagnosed diabetes, liver disease and FPG ≥ 6.1 mmol/L at baseline, as well as subjects who were using medications (including any medication), consuming excessive alcohol at baseline, and those who had incomplete data and withdrew from the survey for unknown reasons. Data from 15,464 subjects were included in the final analysis, and details of the inclusion/exclusion process were shown in Fig. 1.

### Clinical measurement

Subjects' baseline demographic data, drug use, smoking and drinking status, disease history, and exercise habits were collected by standardized questionnaires. Height, weight, WC, systolic blood pressure (SBP), and diastolic blood pressure (DBP) were measured according to established standard methods. BMI was calculated as weight (kg)/[height (m)]^2^; WHtR was calculated as WC (cm)/height (cm); TyG index was calculated as ln [TG (mg/dL) × FPG (mg/dL)/2] [11]; TyG-BMI, TyG-WC, and TyG-WHtR were calculated as multiplying TyG index by BMI, WC, and WHtR [18–21], respectively. Exercise habits were defined as participating in any physical activity more than once a week. Smoking status was defined as none, past, and current based on smoking history. Drinking status was defined as non/small, light, moderate, and heavy according to the weekly alcohol consumption of the subjects in the last month [24].

Venous blood samples for the measurement of biochemical parameters were drawn after the subjects had fasted for at least 8 h, and then alanine aminotransferase (ALT), aspartate aminotransferase (AST), gamma-glutamyl transferase (GGT), glycosylated hemoglobin (HbA1c), FPG, high-density lipoprotein cholesterol (HDL-C), TG, and total cholesterol (TC) concentrations were measured in a standard laboratory using an automated biochemical analyzer.

Fatty liver was determined by color Doppler ultrasound. After the technician performed the ultrasound examination, the gastroenterologist will score and make a diagnosis based on the four sonograms of deep attenuation, hepatorenal echo contrast, vascular blurring, and liver brightness under abdominal color Doppler ultrasound [25].

### Diabetes diagnosis

Diabetes was diagnosed with reference to the American Diabetes Association guidelines [26], including self-reported diabetes in subjects during follow-up and HbA1c ≥ 6.5% or FPG ≥ 7.0 mmol/L measured during follow-up.

### Statistical analysis

We performed all statistical analyses using R language 3.4.3 and EmpowerStats 4.1 software, and statistical significance for all analyses was set at *P* < 0.05 (two-tailed). Subjects were divided into two groups according to whether diabetes occurred during follow-up, and all baseline data were expressed as median (interquartile range), mean [standard deviation (SD)], or frequency (%). The inverse probability of treatment weighting method was used to calculate the weighted standardized difference to quantitatively assess the difference between the two groups, and significance was set at a standardized difference value > 10% [27, 28].

Multivariate Cox proportional hazard regression models were used to estimate HRs and 95% confidence intervals (CIs) for diabetes risk associated with the TyG index and TyG-related parameters, and confounders adjusted in the model were selected according to the epidemiology of diabetes [29]. To standardize the HR corresponding to the TyG index and TyG-related parameters, we performed a Z-transformation on each of these parameters and expressed the final results uniformly as the HR associated with Per SD. First, an Unadjusted Model was established, and then the demographic factors (sex, age, and height) were adjusted in Model I; Model II further considered the influence of blood glucose-, blood lipid-, and blood pressure-related parameters on the risk of diabetes (sex, age, height, TC, HDL-C, HbA1c, SBP); Model III additionally adjusted for exercise habits, smoking status, drinking status, and fatty liver, which have important effects on the incidence of diabetes, on the basis of model II. To test whether the proportional hazards assumption in the Cox regression models was satisfied and whether the covariates were properly adjusted, we used the Kaplan–Meier method and the log-rank test to verify the proportional hazards assumption [30], as well as multiple linear regression analysis to assess the collinearity of the TyG index and TyG-related parameters with other covariates, and covariates with a variance inflation factor greater than 5 were considered collinear variables [31].

To verify the stability of the main results, we performed several sensitivity analyses based on Model III in different subject populations. The first sensitivity analysis further adjusted for liver enzyme-related variables (ALT, AST, and GGT) in all subjects; to reduce potential lagged effects and reverse causality, the second sensitivity analysis included only subjects who were followed up for more than 2 years. In the third and fourth sensitivity analysis, we excluded fatty liver patients and overweight/obese subjects who were prone to diabetes, respectively. Finally, based on Model III we also assessed the possible effect of unmeasured confounders on the association between the TyG index and TyG-related parameters and diabetes risk by calculating the E-value of the TyG index and TyG-related parameters; the E-value quantified the magnitude of the need for unmeasured confounders to negate the association between TyG-related parameters and diabetes risk [32].

In order to further evaluate and compare the predictive value of the baseline TyG index and TyG-related parameters for the onset of diabetes in different future periods, we constructed time-dependent ROC curves for all of the above parameters for predicting the onset of diabetes at years 2–12 of follow-up and calculated the AUCs and predictive thresholds for the corresponding parameters at each time point.

## Results

### Description of subject's baseline information

In the screening procedure in Fig. 1, we excluded 323 subjects with diagnosed diabetes at baseline, 416 subjects with diagnosed liver disease (except fatty liver) at baseline, 739 subjects with excessive alcohol consumption, 808 subjects with FPG ≥ 6.1 mmol/L at baseline, 10 subjects who withdrew from the follow-up cohort for unknown reasons, 863 subjects with incomplete data, and 2321 subjects taking medication (including any medication) at baseline on the basis of the initial 20,944 subjects; a total of 15,464 subjects were eventually enrolled in the study, of which 8430 were men and 7034 were women. The total incidence of diabetes in the current cohort was 39.88/10,000 person-years, including 54.82/10,000 person-years for men and 21.03/10,000 person-years for women. Table 1 depicts baseline information for subjects who developed diabetes during follow-up and those who did not, respectively, and we found that subjects in the diabetic group were more likely to be men, smokers, drinkers, non-exercisers, and fatty liver patients than those in the non-diabetic group; and they were usually older, with higher height, weight, BMI, WC, WHtR, TyG-index, TyG-BMI, TyG-WC, TyG-WHtR, ALT, AST, GGT, TC, TG, FPG, HbA1c, SBP, DBP levels, and lower HDL-C levels (all standardized difference > 10%). By examining and comparing the standardized difference values ​​between the two groups, we found that among all the baseline indicators, the non-diabetic group and the diabetic group had the largest differences in blood glucose-related parameters (FPG and HbA1c) and TyG index and TyG-related parameters (all standardized difference > 94%).

**Table 1:** Baseline demographic, lifestyle, and laboratory characteristics in participants with and without diabetes

	Non-diabetic	Diabetic	Standardized difference, % (95% CI)
Subjects, n	15091	373	
Sex			49 (39, 59)
Women	6947 (46.03%)	87 (23.32%)	
Men	8144 (53.97%)	286 (76.68%)	
Age, years	42.00 (37.00–50.00)	46.00 (41.00–53.00)	40 (30, 51)
Height, m	1.65 (0.08)	1.67 (0.09)	19 (9, 29)
Weight, kg	60.41 (11.48)	69.84 (13.32)	76 (65, 86)
BMI, kg/m^2^	22.04 (3.07)	25.03 (3.82)	86 (76, 97)
WC, cm	76.26 (8.97)	85.08 (10.20)	92 (82, 102)
WHtR	0.46 (0.05)	0.51 (0.06)	90 (80, 100)
TyG-index	8.02 (0.64)	8.62 (0.64)	94 (84, 104)
TyG-BMI	177.66 (33.86)	216.48 (40.09)	105 (94, 115)
TyG-WC	614.35 (105.95)	735.51 (115.82)	109 (99, 120)
TyG-WHtR	3.72 (0.59)	4.41 (0.66)	111 (101, 122)
ALT, U/L	17.00 (13.00–23.00)	24.00 (18.00–39.00)	67 (56, 77)
AST, U/L	17.00 (14.00–21.00)	20.00 (16.00–26.00)	44 (34, 55)
GGT, U/L	15.00 (11.00–22.00)	24.00 (17.00–36.00)	47 (37, 58)
HDL-C, mmol/L	1.42 (1.17–1.71)	1.13 (0.96–1.32)	77 (66, 87)
TC, mmol/L	5.12 (0.86)	5.43 (0.90)	35 (25, 46)
TG, mmol/L	0.72 (0.49–1.11)	1.21 (0.86–1.93)	73 (62, 83)
FPG, mmol/L	5.15 (0.41)	5.61 (0.36)	121 (111, 132)
HbA1c, %	5.16 (0.32)	5.53 (0.37)	107 (97, 118)
SBP, mmHg	114.31 (14.91)	122.03 (15.59)	51 (40, 61)
DBP, mmHg	71.44 (10.47)	77.18 (10.23)	55 (45, 66)
Fatty liver	2518 (16.69%)	223 (59.79%)	99 (89, 109)
Exercise habits	2658 (17.61%)	51 (13.67%)	11 (1, 21)
Drinking status			21 (11, 31)
Non/small	11539 (76.46%)	266 (71.31%)	
Light	1718 (11.38%)	40 (10.72%)	
Moderate	1323 (8.77%)	37 (9.92%)	
Heavy	511 (3.39%)	30 (8.04%)	
Smoking status			45 (35, 55)
None	8886 (58.88%)	145 (38.87%)	
Past	2875 (19.05%)	77 (20.64%)	
Current	3330 (22.07%)	151 (40.48%)	

Values were expressed as mean (SD) or medians (quartile interval) or n (%)

*BMI* Body mass index, *WC* Waist circumference, *WHtR* Waist-to-height ratio, *TyG index *Triglyceride-glucose index, *TyG-BMI* Triglyceride glucose-body mass index, *TyG-WC* Triglyceride glucose-waist circumference, *TyG-WHtR* Triglyceride glucose-waist-to-height ratio, *ALT* Alanine aminotransferase, *AST*: Aspartate aminotransferase, *GGT* Gamma-glutamyl transferase, *HDL-C* High-density lipoprotein cholesterol, *TC* Total cholesterol, *TG* Triglyceride, *HbA1c* Glycated hemoglobin A1c, *FPG* Fasting plasma glucose, *SBP* Systolic blood pressure, *DBP* Diastolic blood pressure

### Comparison of the TyG index and TyG-related parameters in diabetes risk assessment

Before performing multivariable-adjusted Cox regression analysis, it was observed that the proportional hazards assumption was appropriate (Additional file 1: Figs. S1–S4) and the collinearity screening excluded the covariates that were collinear with the TyG index and TyG-related parameters, namely weight, BMI, WC, WHtR, TG, and DBP (Additional file 2: Tables S1–S4). In addition, given that the TyG index and TyG-related parameters have different measurement scales, it may not be appropriate to directly compare the HR values ​​for diabetes risk associated with changes in each unit of them. Therefore, before incorporating them into the Cox regression models, we carried out Z-transformations of the TyG index and TyG-related parameters to obtain the standardized HR values of the corresponding parameters, and then compared their ability to assess the risk of diabetes (Table 2). In the univariate Cox regression model, the TyG index and TyG-related parameters were strongly correlated with the risk of diabetes (HRs: 2.29–2.67); while after further adjustment of demographic and anthropometric indicators (sex, age, and height), blood glucose, blood lipid, and blood pressure parameters (TC, HDL-C, HbA1c, and SBP), as well as living habits and fatty liver, TyG index and TyG-related parameters still maintained a significantly positive correlation with the risk of diabetes. Among them, TyG-WC had the strongest correlation with diabetes risk (HR per SD increase: 1.70, 95% CI 1.46, 1.97), followed by TyG-WHtR (HR per SD increase: 1.63, 95% CI 1.42, 1.87) and TyG-BMI (HR per SD increase: 1.51, 95% CI 1.32, 1.72), however, the correlation between the TyG index and diabetes risk was relatively weak (HR per SD increase: 1.33, 95% CI 1.14, 1.55).

**Table 2:** Association of baseline TyG index and TyG-related parameters with future risk of diabetes

	HR (95% CI)			
	Unadjusted Model	Model I	Model II	Model III
TyG index (Per SD increase)	2.35 (2.13, 2.60)	2.19 (1.96, 2.45)	1.50 (1.29, 1.75)	1.33 (1.14, 1.55)
TyG-BMI (Per SD increase)	2.29 (2.13, 2.47)	2.38 (2.18, 2.59)	1.75 (1.55, 1.98)	1.51 (1.32, 1.72)
TyG-WC (Per SD increase)	2.58 (2.36, 2.83)	**2.78 (2.50, 3.08)**	**2.02 (1.76, 2.32)**	**1.70 (1.46, 1.97)**
TyG-WHtR (Per SD increase)	**2.67 (2.44, 2.92)**	2.57 (2.34, 2.82)	1.92 (1.69, 2.18)	1.63 (1.42, 1.87)

*HR* Hazard ratios, *CI* Confidence interval, other abbreviations as in Table ​1

Model I was adjusted for sex, age, and height

Model II was adjusted for sex, age, height, TC, HDL-C, HbA1c, and SBP

Model III was adjusted for sex, age, height, TC, HDL-C, HbA1c, SBP, Fatty liver, exercise habits, drinking status, and smoking status

### Sensitivity analysis

In order to verify the stability of the results of the above correlation analysis in different populations and exclude the effect of potential reverse causality on the results, based on Model III, we further considered the possible effect of liver enzyme-related parameters (ALT, AST, and GGT) on the risk of diabetes in Sensitivity-1, and excluded subjects with a follow-up period of less than 2 years, fatty liver diagnosed at baseline, and overweight/obesity in Sensitivity 2–4, respectively. Obviously, the results shown in Table 3 proved that the associations between the TyG index and TyG-related parameters and the risk of diabetes were very stable. In these four sensitivity analyses, the direction and magnitude of associations between the TyG index and TyG-related parameters and diabetes risk remained the same as the main analysis, that was, TyG-WC > TyG-WHtR > TyG-BMI > TyG index. Additionally, we calculated the E-value of the TyG index and TyG-related parameters to assess the possible influence of unmeasured factors on the associations. The results showed that after adjustment based on Model III, the HR values for the risk of diabetes associated with per SD of TyG index, TyG-BMI, TyG-WC, and TyG-WHtR were 1.33, 1.51, 1.70, and 1.63, respectively, while the corresponding E-values were 1.99, 2.39, 2.79, and 2.64, respectively. Following the suggestions of VanderWeele and Ding [32], since the E-values corresponding to the TyG index and TyG-related parameters were relatively large in the current study, this suggested that there was unlikely to be an unmeasured confounder to influence the stability of the association between TyG index and TyG-related parameters and diabetes risk.

**Table 3:** Sensitivity analysis: adjusted hazard ratios and 95% confidence intervals for future risk of diabetes associated with baseline TyG index and TyG-related parameters in different test populations

	HR (95% CI)			
	Sensitivity-1	Sensitivity-2	Sensitivity-3	Sensitivity-4
TyG index (per SD increase)	1.30 (1.11, 1.52)	1.34 (1.13, 1.59)	1.36 (1.08, 1.73)	1.42 (1.15, 1.75)
TyG-BMI (per SD increase)	1.47 (1.29, 1.68)	1.58 (1.37, 1.81)	1.37 (1.08, 1.74)	1.33 (1.00, 1.78)
TyG-WC (per SD increase)	**1.65 (1.42, 1.92)**	**1.78 (1.52, 2.09)**	**1.39 (1.07, 1.81)**	**1.61 (1.23, 2.11)**
TyG-WHtR (per SD increase)	1.59 (1.39, 1.83)	1.71 (1.48, 1.98)	1.37 (1.08, 1.74)	1.56 (1.22, 2.00)

*HR* hazard ratios, *CI* confidence interval, other abbreviations as in Table ​1;

(1) Sensitivity-1: Further adjustment of liver enzyme-related variables (n = 15,464)

(2) Sensitivity-2: Participants with a follow-up of less than 2 years were excluded (n = 12,823)

(3) sensitivity-3: Participants diagnosed with the fatty liver at baseline were excluded (n = 12,723)

(4) sensitivity-4: Participants with BMI ≥ 25 kg/m^2^ at baseline were excluded (n = 12,940);

Sensitivity-1 adjusted for sex, age, height, TC, HDL-C, HbA1c, SBP, Fatty liver, exercise habits, drinking status, smoking status, ALT, AST, and GGT

Sensitivity-2 adjusted for sex, age, height, TC, HDL-C, HbA1c, SBP, Fatty liver, exercise habits, drinking status, and smoking status. Sensitivity-3 adjusted for sex, age, height, TC, HDL-C, HbA1c, SBP, exercise habits, drinking status, and smoking status

Sensitivity-4 adjusted for sex, age, height, TC, HDL-C, HbA1c, SBP, Fatty liver, exercise habits, drinking status, and smoking status

### Time-dependent ROC analysis of the TyG index and TyG-related parameters for predicting the onset of diabetes in different future periods

**Fig. 2:**
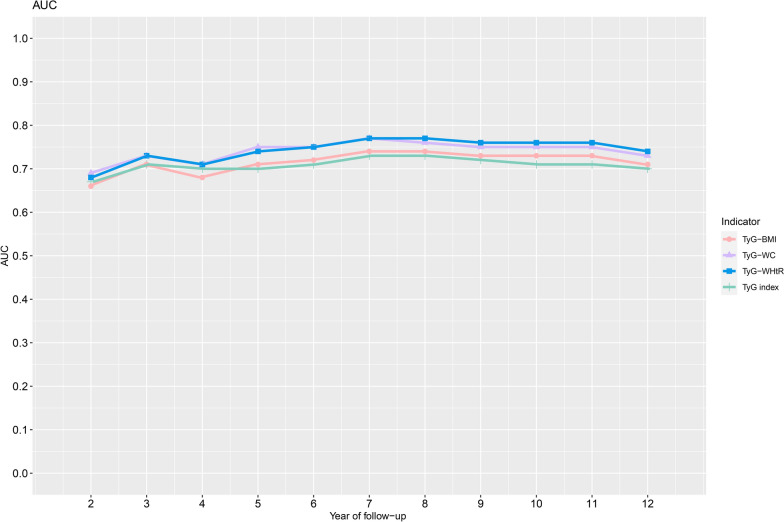
AUC fluctuations of the TyG index, TyG-BMI, TyG-WC, and TyG-WHtR for predicting the onset of diabetes. *AUC* Area under the curves, *TyG index* Triglyceride-glucose index; *TyG-BMI *Triglyceride glucose-body mass index, *TyG-WC* Triglyceride glucose-waist circumference, *TyG-WHtR* Triglyceride glucose- waist-to-height ratio

The AUCs and the prediction thresholds of the TyG index and TyG-related parameters for predicting the onset of diabetes in the next 2–12 years were summarized in Table 4, and to visualize the change in the predictive power of the above indicators over time, the fluctuation curves of the AUC values were also plotted in Fig. 2. Overall, the baseline TyG index and TyG-related parameters had good predictive power for the occurrence of diabetes at different time points in the future, and the AUCs of TyG-related parameters were higher at most time points compared with the TyG index alone. Specifically, by observing Table 4 and the AUC value fluctuation curves (Fig. 2), we found that TyG-WC had the highest predictive accuracy in predicting the onset of diabetes in the short-term (2–6 years), with an AUC value fluctuation curve slightly higher than TyG-WHtR and evidently higher than TyG-BMI and TyG index. As for predicting the occurrence of diabetes in the medium- to long-term future (6–12 years), the AUC value fluctuation curves of the TyG index and TyG-related parameters showed a more evident gap (Fig. 2), with TyG-WHtR having significantly higher predictive accuracy throughout, followed by TyG-WC and TyG-BMI in that order, while the TyG index was relatively weaker in predicting medium- to long-term diabetes risk. In addition, by observing the prediction thresholds of the TyG index and TyG-related parameters in Table 4, we found that the thresholds of TyG-WHtR and TyG index for predicting the onset of diabetes in the next 2–12 years were relatively stable (TyG-WHtR: 3.78–4.07; TyG index: 8.20–8.46), while the threshold fluctuations of TyG-BMI and TyG-WC for the prediction of diabetes in the next 2–12 years were relatively large (TyG-BMI: 159.68–210.13; TyG-WC: 615.38–694.08).

**Table 4:** Best threshold and areas under the time-dependent receiver operating characteristic curves for each baseline TyG index and TyG-related parameters predicting future diabetes risk

Predict time	AUC (best threshold)			
	TyG index	TyG-BMI	TyG-WC	TyG-WHtR
2-years	0.67(8.46)	0.66 (159.68)	**0.69 (622.86)**	0.68 (3.78)
3-years	0.71(8.43)	0.71 (210.13)	**0.73 (632.75)**	0.73 (3.80)
4-years	0.70 (8.23)	0.68 (203.24)	**0.71 (622.86)**	0.71 (4.06)
5-years	0.70 (8.23)	0.71 (201.41)	**0.75 (682.71)**	0.74 (4.07)
6-years	0.71 (8.23)	0.72 (201.41)	0.75 (679.08)	**0.75 (4.05)**
7-years	0.73 (8.23)	0.74 (200.23)	0.77 (684.26)	**0.77 (4.05)**
8-years	0.73 (8.23)	0.74 (200.23)	0.76 (683.33)	**0.77 (4.05)**
9-years	0.72 (8.20)	0.73 (196.49)	0.75 (694.08)	**0.76 (4.05)**
10-years	0.71 (8.21)	0.73 (196.49)	0.75 (679.46)	**0.76 (4.00)**
11-years	0.71 (8.20)	0.73 (182.89)	0.75 (646.24)	**0.76 (3.92)**
12-years	0.70 (8.21)	0.71 (182.60)	0.73 (615.38)	**0.74 (3.92)**

*AUC* area under the curve, other abbreviations as in Table ​1

## Discussion

In this large longitudinal cohort study based on the Japanese physical examination population, we comprehensively and systematically compared the risk assessment/prediction performance of the baseline TyG index and TyG-related parameters for the onset of diabetes in different future periods. Overall, the TyG index and TyG-related parameters were independent predictors of future diabetes risk, and combining the TyG index with obesity parameters BMI, WC, and WHtR can further enhance its ability to assess/predict future diabetes risk. Among them, TyG-WC was not only the best indicator for assessing diabetes risk, but also had the highest predictive accuracy for diabetes risk in the short-term future (2–6 years). However, TyG-WHtR rather than TyG-WC had higher predictive accuracy and more stable prediction thresholds for predicting the onset of diabetes in the medium- to long-term future (6–12 years).

Diabetes, a chronic metabolic disorder disease, is almost incurable but can be effectively prevented by early interventions [1, 4]. The latest tenth edition of the International Diabetes Federation Diabetes Atlas showed that there are approximately 537 million diabetics worldwide, and more than 6.7 million patients aged 20–79 have died from diabetes-related causes, yet it is even more disturbing that 45% of people with diabetes are still undiagnosed and that more and more children and adolescents under the age of 19 are becoming diabetic, the vast majority of whom have type 2 diabetes [33]. It is well known that the core pathophysiological mechanism of type 2 diabetes is IR, which is often present before the onset of diabetes, and therefore screening for the presence of IR is key to diabetes prevention [7, 34].

### Comparison with published data

#### The ability of the TyG index and TyG-related parameters to assess the risk of diabetes

TyG index, a recognized alternative marker for IR, is now widely used in epidemiological investigations and clinical studies of IR-related diseases because it not only has a high sensitivity similar to the gold standard measurement of IR, the hyperinsulinemic-euglycemic clamp technique, but also avoids the invasiveness of the gold standard measurement, additionally, its assessment results are not affected by the accuracy of fasting insulin measurements [11–16, 35]. Correspondingly, the association between the TyG index and diabetes has also been confirmed in a large number of observational studies. For example, in the study by da Silva et al. they performed a meta-analysis of 13 cohort studies involving 70,380 adult subjects on the TyG index predicting type 2 diabetes, and the results showed that the TyG index was significantly positively associated with the risk of type 2 diabetes (overall HR: 2.44, 95% CI 2.17, 2.76) [36]; furthermore, in a study by Navarro-González et al. of 4820 patients in the Vascular Metabolic CUN cohort, the TyG index was found to be a better predictor of the development of type 2 diabetes than FPG and TG alone [37]. Consistent with previous studies, the current study also found that the TyG index was significantly and positively correlated with diabetes risk (HR per SD increase: 1.33, 95% CI 1.14, 1.55) and was an independent predictor of the future development of diabetes. The superior performance of the TyG index in risk assessment and prediction of diabetes may be related to the fact that it explains the effects of both blood glucose and lipid metabolism on β-cell function [38]. Previous studies have shown that chronic hyperglycemia in humans can lead to increased oxidative stress in pancreatic islet tissue [39], and that islet tissue has the lowest antioxidant capacity compared to other metabolic tissues such as liver tissue, adipose tissue, and skeletal muscle because it has less activity of antioxidant enzymes [40, 41]. Therefore, the glucotoxicity of long-term high glucose concentration to pancreatic β cells may be one of the main reasons for the continuous decline in pancreatic islet function and consequently IR [42]. Furthermore, excessive TG deposition in islet cells can also impair the function of islet β cells, and TG levels in target organs such as the liver and skeletal muscle are also considered to be important determinants of hepatic IR and peripheral IR [42, 43]. Evidence from experimental studies suggested that long-term deposition of TG in skeletal muscle can lead to attenuation of skeletal muscle mass and function [44]. Skeletal muscle is the major organ for glucose uptake and utilization in the body, accounting for about 85% of all insulin-mediated glucose utilization, and its myofibers can also express and release cytokines or peptides that are important for maintaining insulin sensitivity of muscle tissue [45, 46]. Therefore, muscle attenuation caused by higher levels of TG in skeletal muscle may also be one of the decisive factors for IR. In addition to the effects of metabolic factors, body fat content and the distribution pattern of fat are also considered to be closely related to the development of IR and diabetes [47, 48], and therefore an increasing number of researchers have been investigating whether TyG index combined with obesity parameters (BMI, WC, WHtR) can further enhance the risk assessment/predictive ability of TyG index for IR-related metabolic diseases such as diabetes and which TyG-related parameters are the best risk markers for screening diabetes, but the results of several published comparative studies are highly controversial [49–51].

In a cross-sectional survey of a normal-weight Chinese elderly population by Ke et al. the TyG index and TyG-related parameters were compared for the risk assessment ability of type 2 diabetes, and it was found that combining the TyG index with BMI, WC, and WHtR could not further improve the role of TyG index in evaluating the occurrence and development of type 2 diabetes [49]; while in another cross-sectional comparative study, Zheng et al. found that among the TyG index and TyG-related parameters, TyG-WC had the strongest correlation with diabetes risk, but the TyG index alone was more powerful than TyG-BMI in assessing the risk of diabetes [50]. In addition, in a cohort study by Li X et al. they simultaneously compared the ability of the TyG index and TyG-BMI, TyG-WC, and TyG-WHtR to assess the risk of diabetes under different glycemic states in the Chinese middle-aged and elderly population, and showed that TyG-related parameters were better than TyG index for the assessment of the risk of developing diabetes in the future, and TyG-BMI was a more important risk factor for developing diabetes regardless of glycemic state [51].

The current study found that TyG-BMI, TyG-WC, and TyG-WHtR were all stronger than the TyG index alone in assessing the risk of future diabetes after multiple comparisons, which was consistent with the results of the cohort study by Li et al. [51], but the difference was that the results of the current study supported that TyG-WC was the best indicator for assessing the risk of developing diabetes rather than TyG-BMI proposed by Li et al. which may be due to differences in study populations and comparison methods. The follow-up cohort of the current study was composed of 15,464 physically examined individuals aged 18–79 years, and the large sample size and age range avoided, to some extent, selection bias in the subject population and made the results of the current study more realistic. Moreover, another often overlooked issue is that the measurement units and measurement scales of the TyG index and TyG-related parameters are different, and it may not be appropriate to directly compare the HR values of diabetes risk associated with each of their unit changes, so the current study standardized the HR values associated with future diabetes risk for the TyG index and TyG-related parameters before making the comparison. It is worth mentioning that the longitudinal cohort design of the current study further considered the effect of time progression on the associations between the TyG index and TyG-related parameters and diabetes risk compared to cross-sectional comparative studies [49, 50], revealing a definite cause-and-effect relationship between them. In addition, the reason why TyG-BMI, TyG-WC, and TyG-WHtR were stronger in assessing future diabetes risk than the TyG index in the current study may be that they additionally reflected the information on obesity and body fat distribution. It is known that obese populations typically have higher levels of oxidative stress and risk of diabetes compared to the general lean population, and these may be associated with more adipose tissue [52]. Adipose tissue is not only a warehouse for lipid storage in the body but also an active endocrine tissue, especially visceral adipose tissue (VAT), which is mainly distributed in the abdomen [53]. Compared with subcutaneous adipose tissue, excessive VAT is more likely to cause endocrine dysfunction in adipose tissue, resulting in an imbalance in the secretion of proinflammatory adipocytokines and defensive adipocytokines [54]. Moreover, the lipolysis and inflammatory responses of VAT stimulated by catecholamines are more intense, which can release more lipolysis products such as glycerol and fatty acids [55, 56]; while the long-term exposure of pancreatic β cells to high levels of fatty acids will not only damage insulin secretion induced by elevated glucose can also lead to impaired insulin gene expression and islet β-cell death [42]. Anyway, abdominal obesity, which is dominated by visceral fat accumulation, may have a greater impact on the body's glucose metabolism than general obesity and is a more important risk factor for diabetes. Correspondingly, evidence from a large number of observational studies also suggested that the anthropometric measures WC and WHtR, which assess abdominal obesity, were not only significantly associated with diabetes risk independently of BMI, but were also stronger predictors of diabetes than BMI [57, 58]. Therefore, the current study found that TyG-WC and TyG-WHtR consistently had stronger diabetes risk assessment ability than TyG-BMI and TyG index probably because they additionally respond to the effect of visceral obesity on diabetes risk.

#### Predictive value of the baseline TyG index and TyG-related parameters for the onset of diabetes in different future periods

Another innovative finding of the current study is the first report of the temporal differences in the predictive performance of the baseline TyG index and TyG-related parameters on the future occurrence of diabetes and the fluctuations of the prediction thresholds. For the comparison of the predictive value of the TyG index and TyG-related parameters for the future development of diabetes, the results of two published longitudinal cohort studies supported the superior predictive value of TyG-related parameters over the TyG index alone and recommend TyG-WHtR as the best risk marker for predicting the future development of diabetes [51, 59]. It should be noted, however, that the two published studies did not consider the effect of the time factor (time variables were not included in the ROC analysis) when assessing the predictive value of the baseline TyG index and TyG-related parameters, which would not only caused their analyses to lose information on time-related disease states and risk factors [60], but would also prevent them from comparing the predictive accuracy and threshold fluctuations of each parameter for predicting the occurrence of diabetes at different time points in the future [61]. Therefore, to address these issues, we used time-dependent ROC analysis in the current study to assess the fluctuations in predictive accuracy and prediction thresholds of the above parameters for the occurrence of diabetes over the next 2–12 years.

Our results showed that both the TyG index and TyG-related parameters had a good predictive performance for future diabetes (most AUC values were above 0.7), that the predictive accuracy of these parameters tended to increase and then decrease with the extension of follow-up time, and that the AUC values of the TyG-related parameters were higher than those of the TyG index alone at most time points. On this basis, we further explored the best risk markers for predicting the onset of diabetes at different periods in the future. We found that TyG-WC was more accurate than TyG-WHtR, TyG-BMI, and TyG index in predicting diabetes in the short-term future (2–6 years), but with the further extension of follow-up time, the power of TyG-WHtR to predict diabetes gradually increased and surpassed TyG-WC after year 6, becoming the best risk marker for predicting the occurrence of diabetes in the future medium- to long-term (6–12 years). It is worth mentioning that in the threshold analysis, we also found that the thresholds of TyG-WHtR for diabetes prediction fluctuated less, especially after year 4 of follow-up when its prediction threshold was almost constant, implying that TyG-WHtR was a stable predictor of diabetes risk in the medium- to long-term future. In summary, TyG-WC had the strongest risk assessment ability for the onset of diabetes and the highest predictive performance for short-term (2–6 years) diabetes risk, while TyG-WHtR was more suitable for predicting future diabetes risk in the medium- to long-term (6–12 years), and therefore the joint assessment of TyG-WC and TyG-WHtR in primary health care may be the best strategy for diabetes prevention.

#### Implications of this study

The current study comprehensively compared the risk assessment/predictive value of the TyG index and TyG-related parameters for diabetes in a larger Japanese population using standardized and rigorous statistical methods. We demonstrated the value of TyG-related parameters in risk assessment/prediction of diabetes over the TyG index alone and recommend TyG-WC as the primary surveillance parameter for diabetes screening and clinical assessment/prediction of diabetes risk in large populations, and TyG-WHtR as the best risk marker for predicting future risk of diabetes in the medium- to long-term; therefore, simultaneous assessment of TyG-WC and TyG-WHtR levels may be of greater clinical value. The current study’s findings resolved the debate about whether the TyG index combined with obesity parameters further enhances its risk assessment/predictive power for diabetes and provided more relevant and practical references for diabetes screening and prevention.

#### Strengths and limitations

The strengths of the current study are the following: (1) The current study included 15,464 subjects aged 18–79 who were examined and followed up for a long period of time (up to 13 years) and further employment of time-dependent ROC analysis on this basis allowed us to more comprehensively compare the predictive value of the baseline TyG index and TyG-related parameters, TyG-BMI, TyG-WC, and TyG-WHtR, for the occurrence of diabetes at different time points in the future. To my knowledge, the current study is the first to simultaneously compare the risk assessment/predictive value of the baseline TyG index and TyG-related parameters for the future development of diabetes in a large sample population, and the first to find temporal differences and threshold fluctuations in the predictive performance of the above parameters for the risk of future diabetes. (2) The findings of the current study are relatively reliable as several sensitivity analyses and adequate model adjustments were performed and the HR values of the TyG index and TyG-related parameters associated with diabetes risk were standardized before the comparisons.

This study also has some limitations: (1) The original dataset of the current study did not contain information on the fasting insulin levels of the subjects, so we cannot further compare the risk assessment/predictive ability of the TyG index and TyG-related parameters with HOMA-IR for the future development of diabetes in the current study cohort. (2) The outcome of interest in the current study was diabetes, but the diagnostic criteria did not include the subject's 2-h postprandial blood glucose level, which may lead us to underestimate the incidence of diabetes [62, 63]. (3) The current study did not differentiate between types of diabetes, but with reference to epidemiological surveys of diabetes and previous studies of the TyG index and TyG-related parameters, the results of the present study may be more applicable to type 2 diabetes [1–3, 64]. (4) Although risk factors for diabetes have been adequately adjusted for in the current study, there were still variables such as dietary habits and women’s reproductive status that were not included in the model adjustment due to secondary analysis of data from previous studies, and these unmeasured factors may affect the predictive power of TyG-related parameters and lead to residual confounding [65]. To verify the effect of unmeasured factors on the current study, we calculated E-value for TyG-related parameters, and the results indicated that the findings of the current study are relatively robust. (5) The current study did not repeatedly measure the TyG index and TyG-related parameters in subjects during follow-up, and therefore the possible impact of dynamic changes in TyG-related parameters on the risk assessment/predictive power for future diabetes could not be explored and needs to be further explored in future studies. (6) The current study is a single-center cohort study based on a Japanese population, so the applicability of the results of the current study to other ethnic/national populations needs to be validated by further studies. In addition, the subjects in the current study were those who underwent health checkups, and this population may have better health awareness and physical condition than the general population, so the current study may not be representative of the results in the general population, and further validation in the general population is needed. (7) Since the subjects in the current cohort study were not clearly differentiated into exposed and non-exposed groups or screening intervention and non-screening intervention groups at baseline and during follow-up, the results of the current study reported only relative risk estimates for the TyG index and TyG-related parameters, but not absolute risk estimates that may be more clinically meaningful.

## Conclusions

With regard to the prevention and management of chronic diseases, the exploration of simple and reliable risk markers has always been very important. The results of this study supported the superiority of TyG-related parameters over the TyG index alone for risk assessment/prediction of future diabetes; moreover, after a comprehensive assessment of TyG-related parameters, we recommend TyG-WC as a clinically valid marker for assessing and predicting short-term diabetes risk, on the basis of which further assessment of TyG-WHtR levels will help predict future diabetes risk in the medium- to long-term.

## Supplementary Information

**Additional file 1: Figure S1.** Kaplan-meier curve of TyG index quartiles over time. TyG index: triglyceride-glucose index. **Figure S2.** Kaplan-meier curve of TyG-BMI quartiles over time. TyG-BMI: triglyceride glucose-body mass index. **Figure S3.** Kaplan-meier curve of TyG-WC quartiles over time. TyG-WC: triglyceride glucose-waist circumference. **Figure S4.** Kaplan-meier curve of TyG-WHtR quartiles over time. TyG-WHtR: triglyceride glucose- waist-to-height ratio.

**Additional file 2: Table S1.** Collinearity diagnostics steps of TyG index with other covariates. **Table S2.** Collinearity diagnostics steps of TyG-BMI with other covariates. **Table S3.** Collinearity diagnostics steps of TyG-WC with other covariates. **Table S4.** Collinearity diagnostics steps of TyG-WHtR with other covariates.

## Data Availability

The data used in this study have been uploaded to the "Dryad" database by Professor Okamura et al. (https://datadryad.org/stash/dataset/doi:10.5061/dryad.8q0p192).

## Abbreviations

TyG Index: Triglyceride-glucose index
TyG-BMI: Triglyceride glucose-body mass index
TyG-WC: Triglyceride glucose-waist circumference
TyG-WHtR: Triglyceride glucose-waist to height ratio
ALT: Alanine aminotransferase
AST: Aspartate aminotransferase
GGT: Gamma-glutamyl transferase
HDL-C: High-density lipoprotein cholesterol
TC: Total cholesterol
TG: Triglyceride
HbA1c: Glycated hemoglobin A1c
FPG: Fasting plasma glucose
SBP: Systolic blood pressure
DBP: Diastolic blood pressure
ROC: Receiver operating characteristic
HR: Hazard ratio
CI: Confidence interval
AUC: Area under the curves
SD: Standard deviation
VAT: Visceral adipose tissue
