# Supplementary material for: Assessing temporal differences in the predictive power of baseline TyG-related parameters for future diabetes: an analysis using time-dependent receiver operating characteristics
Source: J Transl Med. 2023 May 4;21:299. doi: 10.1186/s12967-023-04159-7 (PMC10158224; doi:10.1186/s12967-023-04159-7)
Supplement: Supplementary file 1 — Additional file 1: Figure S1. Kaplan-meier curve of TyG index quartiles over time. TyG index: triglyceride-glucose index. Figure S2. Kaplan-meier curve of TyG-BMI quartiles over time. TyG-BMI: triglyceride glucose-body mass index. Figure S3. Kaplan-meier curve of TyG-WC quartiles over time. TyG-WC: triglyceride glucose-waist circumference. Figure S4. Kaplan-meier curve of TyG-WHtR quartiles over time. TyG-WHtR: triglyceride glucose- waist-to-height ratio. [file 12967_2023_4159_MOESM1_ESM.docx]

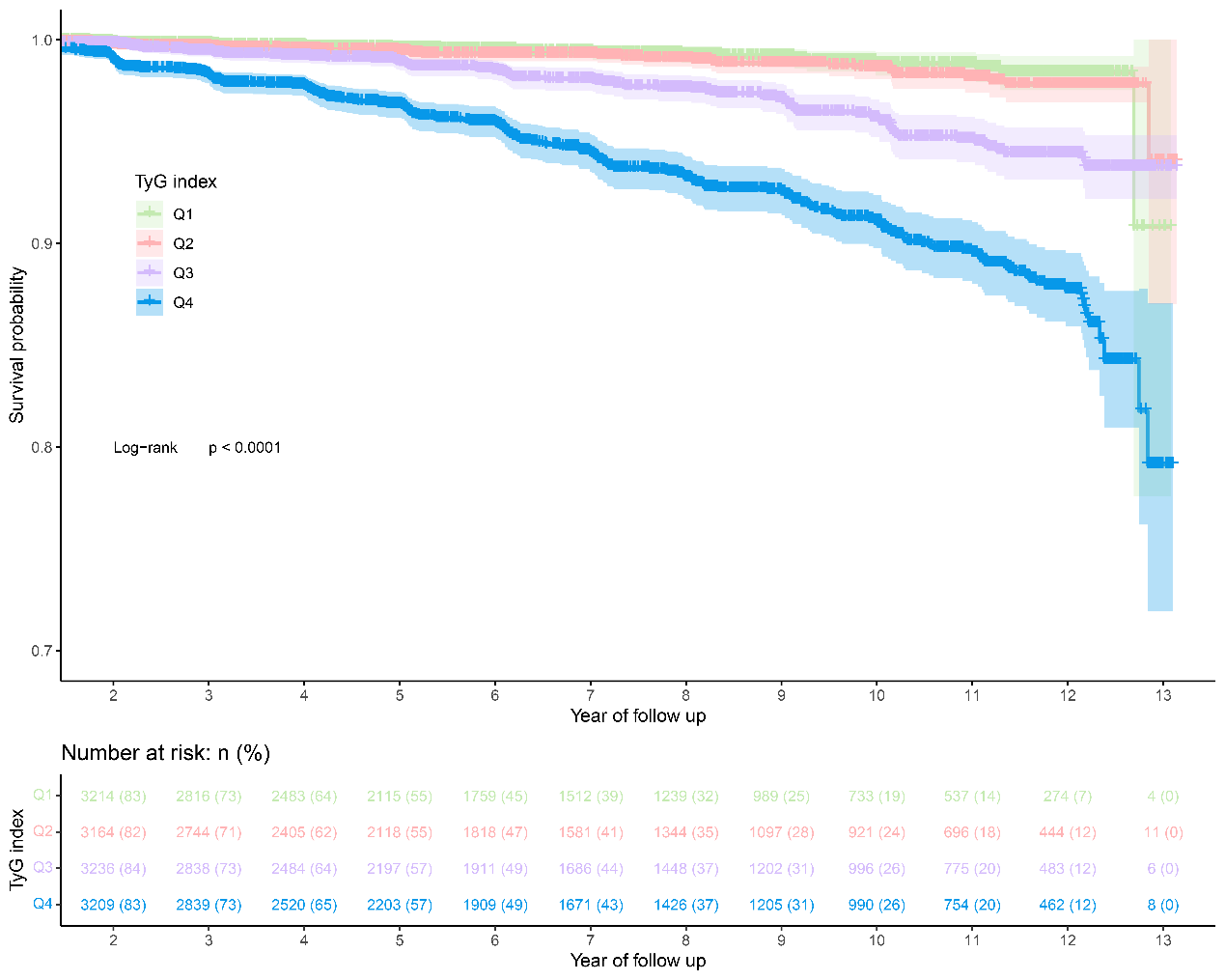


**Supplementary Figure 1:** Kaplan-meier curve of TyG index quartiles over time. TyG index: triglyceride-glucose index.


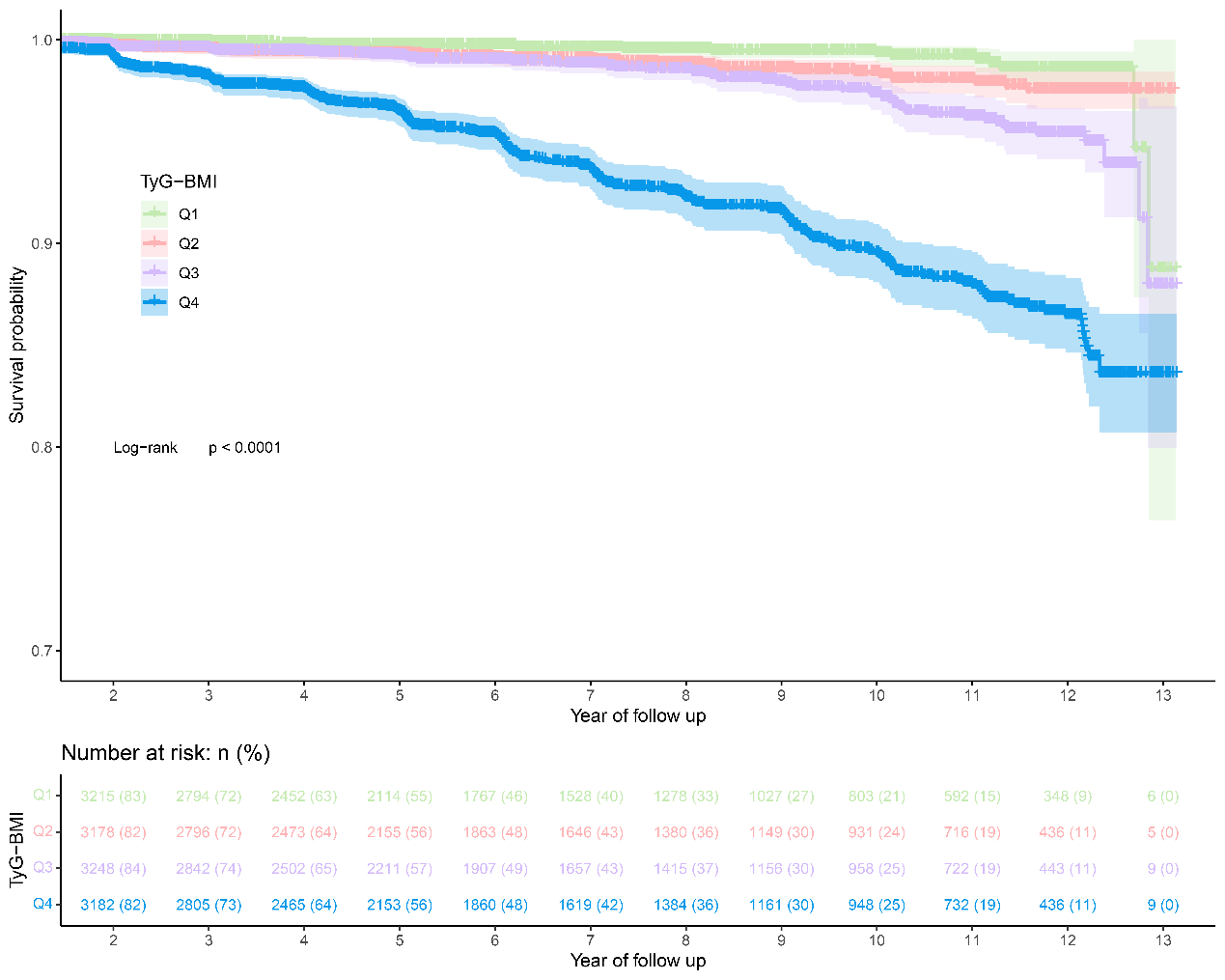


**Supplementary Figure 2:** Kaplan-meier curve of TyG-BMI quartiles over time. TyG-BMI: triglyceride glucose-body mass index.


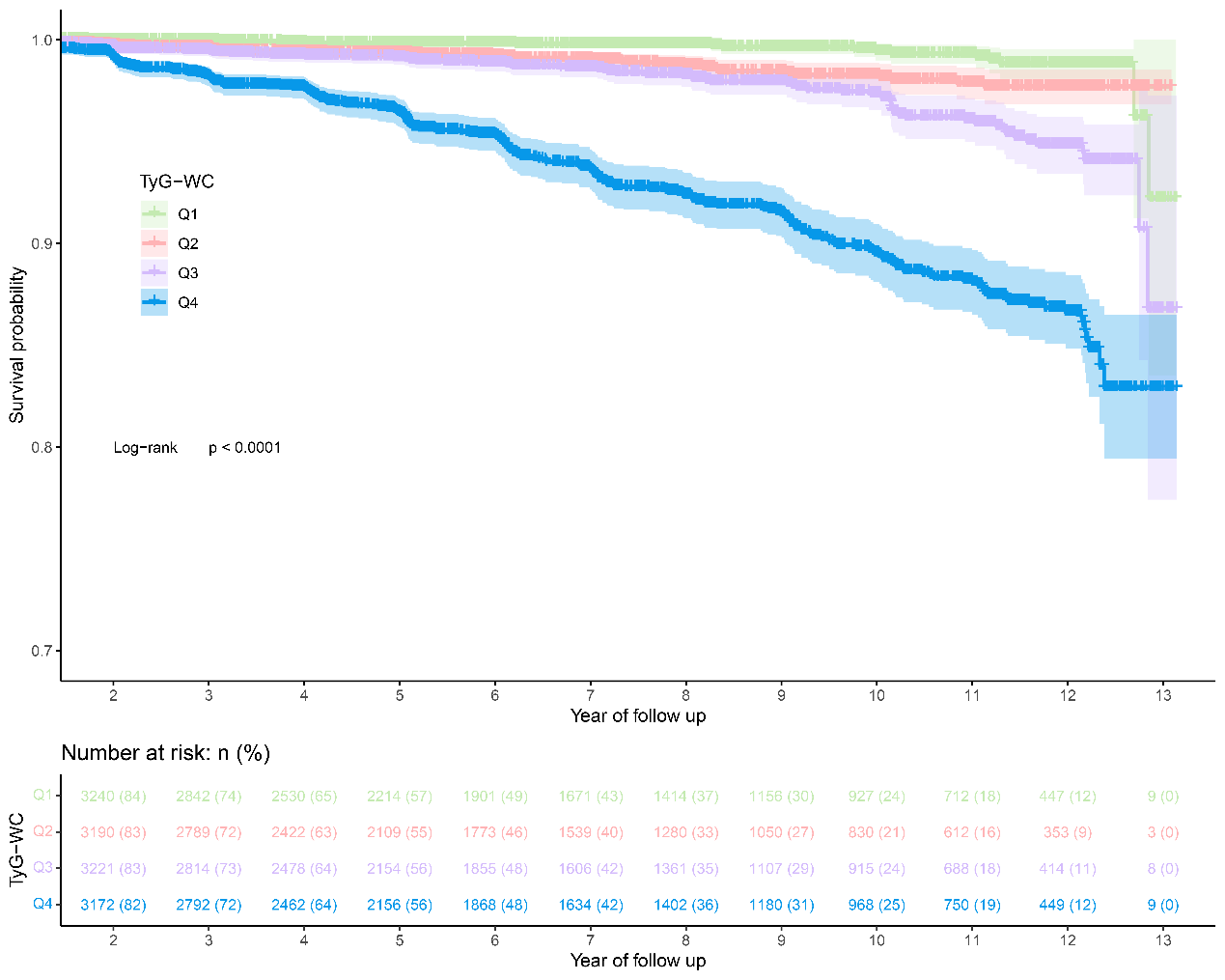


**Supplementary Figure 3:** Kaplan-meier curve of TyG-WC quartiles over time. TyG-WC: triglyceride glucose-waist circumference.


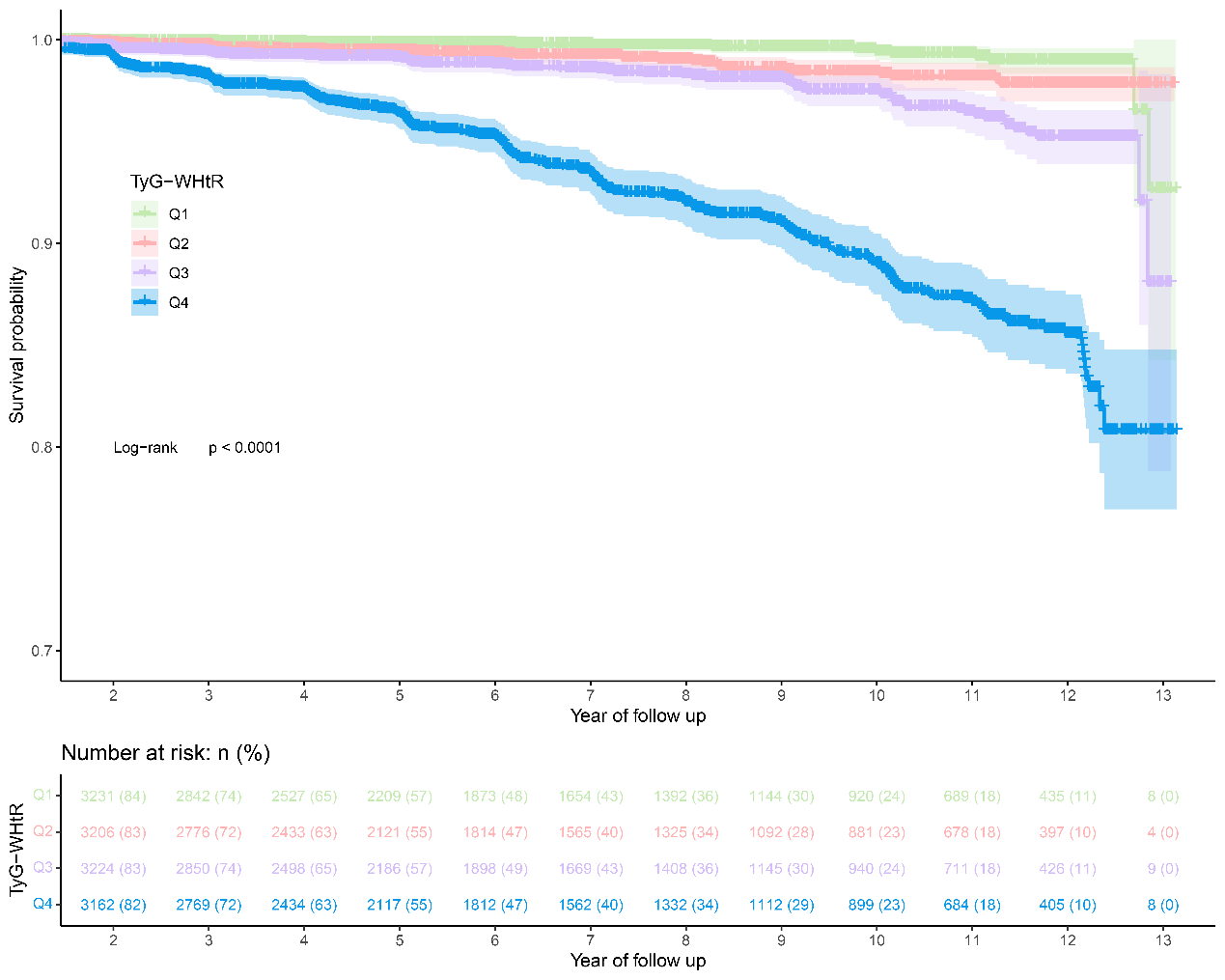


**Supplementary Figure 4:** Kaplan-meier curve of TyG-WHtR quartiles over time. TyG-WHtR: triglyceride glucose- waist-to-height ratio.
