# Supplementary material for: Assessing temporal differences in the predictive power of baseline TyG-related parameters for future diabetes: an analysis using time-dependent receiver operating characteristics
Source: J Transl Med. 2023 May 4;21:299. doi: 10.1186/s12967-023-04159-7 (PMC10158224; doi:10.1186/s12967-023-04159-7)
Supplement: Supplementary file 2 — Additional file 2: Table S1. Collinearity diagnostics steps of TyG index with other covariates. Table S2. Collinearity diagnostics steps of TyG-BMI with other covariates. Table S3. Collinearity diagnostics steps of TyG-WC with other covariates. Table S4. Collinearity diagnostics steps of TyG-WHtR with other covariates. [file 12967_2023_4159_MOESM2_ESM.docx]

Supplementary Table 1: Collinearity diagnostics steps of TyG index with other covariates.

|  | Variance inflation factor | | | | | |
| --- | --- | --- | --- | --- | --- | --- |
|  | Step 1 | Step 2 | Step 3 | Step 4 | Step 5 | Step 6 |
| TyG index | 7 | 7 | 7 | 7 | 2.3 | 2.3 |
| Sex | 3.3 | 3.3 | 3.2 | 3.2 | 3.2 | 3.2 |
| Age | 1.4 | 1.4 | 1.4 | 1.4 | 1.4 | 1.3 |
| Height | 91.1 | 52.3 | 2.5 | 2.5 | 2.5 | 2.4 |
| Weight | 426.2 | 171.1 | NA | NA | NA | NA |
| BMI | 229.1 | 99.9 | 5 | 5 | 5 | NA |
| WC | 1194.5 | NA | NA | NA | NA | NA |
| WHtR | 946.6 | 4.8 | 4.7 | 4.7 | 4.7 | 1.7 |
| ALT | 4.2 | 4.2 | 4.2 | 4.1 | 4.1 | 4.1 |
| AST | 3.3 | 3.3 | 3.3 | 3.3 | 3.3 | 3.3 |
| GGT | 1.5 | 1.5 | 1.5 | 1.5 | 1.5 | 1.5 |
| TC | 1.5 | 1.5 | 1.5 | 1.5 | 1.5 | 1.5 |
| HDL-C | 1.9 | 1.9 | 1.9 | 1.9 | 1.9 | 1.9 |
| TG | 5.4 | 5.3 | 5.3 | 5.3 | NA | NA |
| HbA1c | 1.3 | 1.3 | 1.3 | 1.3 | 1.3 | 1.3 |
| FPG | 1.6 | 1.6 | 1.6 | 1.6 | 1.6 | 1.6 |
| SBP | 5.6 | 5.6 | 5.6 | 1.4 | 1.4 | 1.4 |
| DBP | 5.7 | 5.7 | 5.7 | NA | NA | NA |
| Fatty liver | 1.6 | 1.6 | 1.6 | 1.6 | 1.6 | 1.5 |
| Exercise | 1 | 1 | 1 | 1 | 1 | 1 |
| Drinking status | 1.3 | 1.3 | 1.3 | 1.3 | 1.3 | 1.3 |
| Smoking status | 1.4 | 1.4 | 1.4 | 1.4 | 1.4 | 1.4 |

Abbreviations: Inf: infinity; VIF: Variance inflation factor; Other abbreviations as in Table ​1.

Note-1: Variance inflation factor = 1/(1-R^2^). Abbreviations as in Table 1.

Note-2: The variables with Variance inflation factor >5 will be regarded as collinear variables and cannot be included in the multiple regression model.

Supplementary Table 2: Collinearity diagnostics steps of TyG-BMI with other covariates.

|  | Variance inflation factor | | | | |
| --- | --- | --- | --- | --- | --- |
|  | Step 1 | Step 2 | Step 3 | Step 4 | Step 5 |
| TyG-BMI | 46.6 | 46.6 | 46.5 | 7.5 | 7.4 |
| Sex | 3.2 | 3.2 | 3.2 | 3.2 | 3.2 |
| Age | 1.4 | 1.4 | 1.4 | 1.4 | 1.3 |
| Height | 91.1 | 52.4 | 2.5 | 2.5 | 2.5 |
| Weight | 426.5 | 171.3 | NA | NA | NA |
| BMI | 251.3 | 122 | 30.9 | NA | NA |
| WC | 1194.3 | NA | NA | NA | NA |
| WHtR | 946.3 | 4.8 | 4.7 | 4.2 | 4.2 |
| ALT | 4.2 | 4.2 | 4.2 | 4.2 | 4.1 |
| AST | 3.3 | 3.3 | 3.3 | 3.3 | 3.3 |
| GGT | 1.5 | 1.5 | 1.5 | 1.5 | 1.5 |
| TC | 1.5 | 1.5 | 1.5 | 1.5 | 1.5 |
| HDL-C | 1.9 | 1.9 | 1.9 | 1.9 | 1.9 |
| TG | 6.3 | 6.3 | 6.3 | 2.5 | 2.5 |
| HbA1c | 1.3 | 1.3 | 1.3 | 1.3 | 1.3 |
| FPG | 1.6 | 1.6 | 1.6 | 1.5 | 1.5 |
| SBP | 5.6 | 5.6 | 5.6 | 5.6 | 1.4 |
| DBP | 5.7 | 5.7 | 5.7 | 5.7 | NA |
| Fatty liver | 1.6 | 1.6 | 1.6 | 1.6 | 1.6 |
| Exercise | 1 | 1 | 1 | 1 | 1 |
| Drinking status | 1.3 | 1.3 | 1.3 | 1.3 | 1.3 |
| Smoking status | 1.4 | 1.4 | 1.4 | 1.4 | 1.4 |

Abbreviations: Inf: infinity; VIF: Variance inflation factor; Other abbreviations as in Table ​1.

Note-1: Variance inflation factor = 1/(1-R^2^). Abbreviations as in Table 1.

Note-2: The variables with Variance inflation factor >5 will be regarded as collinear variables and cannot be included in the multiple regression model.

Supplementary Table 3: Collinearity diagnostics steps of TyG-WC with other covariates.

|  | Variance inflation factor | | | | |
| --- | --- | --- | --- | --- | --- |
|  | Step 1 | Step 2 | Step 3 | Step 4 | Step 5 |
| TyG-WC | 38.8 | 38 | 36.6 | 9.7 | 9.7 |
| Sex | 3.3 | 3.2 | 3.2 | 3.2 | 3.2 |
| Age | 1.4 | 1.4 | 1.4 | 1.4 | 1.4 |
| Height | 91.2 | 52.5 | 5.6 | 2.7 | 2.7 |
| Weight | 426.3 | 177.5 | NA | NA | NA |
| BMI | 229.1 | 102.8 | 5 | 4.4 | 4.4 |
| WC | 1220.2 | NA | NA | NA | NA |
| WHtR | 946.9 | 18.5 | 17.7 | NA | NA |
| ALT | 4.2 | 4.2 | 4.2 | 4.1 | 4.1 |
| AST | 3.3 | 3.3 | 3.3 | 3.3 | 3.3 |
| GGT | 1.5 | 1.5 | 1.5 | 1.5 | 1.5 |
| TC | 1.5 | 1.5 | 1.5 | 1.5 | 1.5 |
| HDL-C | 1.9 | 1.9 | 1.9 | 1.9 | 1.9 |
| TG | 6.4 | 6.3 | 6.2 | 3 | 3 |
| HbA1c | 1.3 | 1.3 | 1.3 | 1.3 | 1.3 |
| FPG | 1.7 | 1.7 | 1.6 | 1.5 | 1.5 |
| SBP | 5.6 | 5.6 | 5.6 | 5.6 | 1.4 |
| DBP | 5.7 | 5.7 | 5.7 | 5.7 | NA |
| Fatty liver | 1.6 | 1.6 | 1.6 | 1.6 | 1.6 |
| Exercise | 1 | 1 | 1 | 1 | 1 |
| Drinking status | 1.3 | 1.3 | 1.3 | 1.3 | 1.3 |
| Smoking status | 1.4 | 1.4 | 1.4 | 1.4 | 1.4 |

Abbreviations: Inf: infinity; VIF: Variance inflation factor; Other abbreviations as in Table ​1.

Note-1: Variance inflation factor = 1/(1-R^2^). Abbreviations as in Table 1.

Note-2: The variables with Variance inflation factor >5 will be regarded as collinear variables and cannot be included in the multiple regression model.

Supplementary Table 4: Collinearity diagnostics steps of TyG-WHtR with other covariates.

|  | Variance inflation factor | | | | |
| --- | --- | --- | --- | --- | --- |
|  | Step 1 | Step 2 | Step 3 | Step 4 | Step 5 |
| TyG-WHtR | 31.2 | 31.2 | 31.2 | 7.9 | 7.9 |
| Sex | 3.3 | 3.3 | 3.2 | 3.2 | 3.2 |
| Age | 1.4 | 1.4 | 1.4 | 1.4 | 1.4 |
| Height | 91 | 52.3 | 2.5 | 2.5 | 2.5 |
| Weight | 426.3 | 171 | NA | NA | NA |
| BMI | 229.2 | 99.9 | 5 | 4.3 | 4.3 |
| WC | 1194.4 | NA | NA | NA | NA |
| WHtR | 957.9 | 18.6 | 18.6 | NA | NA |
| ALT | 4.2 | 4.2 | 4.2 | 4.1 | 4.1 |
| AST | 3.3 | 3.3 | 3.3 | 3.3 | 3.3 |
| GGT | 1.5 | 1.5 | 1.5 | 1.5 | 1.5 |
| TC | 1.5 | 1.5 | 1.5 | 1.5 | 1.5 |
| HDL-C | 1.9 | 1.9 | 1.9 | 1.9 | 1.9 |
| TG | 6.1 | 6.1 | 6.1 | 2.9 | 2.9 |
| HbA1c | 1.3 | 1.3 | 1.3 | 1.3 | 1.3 |
| FPG | 1.7 | 1.7 | 1.7 | 1.5 | 1.5 |
| SBP | 5.6 | 5.6 | 5.6 | 5.6 | 1.4 |
| DBP | 5.7 | 5.7 | 5.7 | 5.7 | NA |
| Fatty liver | 1.6 | 1.6 | 1.6 | 1.6 | 1.6 |
| Exercise | 1 | 1 | 1 | 1 | 1 |
| Drinking status | 1.3 | 1.3 | 1.3 | 1.3 | 1.3 |
| Smoking status | 1.4 | 1.4 | 1.4 | 1.4 | 1.4 |

Abbreviations: Inf: infinity; VIF: Variance inflation factor; Other abbreviations as in Table ​1.

Note-1: Variance inflation factor = 1/(1-R^2^). Abbreviations as in Table 1.

Note-2: The variables with Variance inflation factor >5 will be regarded as collinear variables and cannot be included in the multiple regression model.
